# Quinine in Suppuration

**Published:** 1875-03

**Authors:** Douglas Morton

**Affiliations:** Physician to the Louisville (Kentucky) Hospital


### Selections.

### QUININE IN SUPPURATION.

BY DOUGLAS MORTON, A.M., M.D.,

*Physician to the Louisville (Kentucky) Hospital.*

Some months since—before I had given any special attention to the property lately discovered in quinine of exercising control over the suppurative process—a friend gave me a formula containing this agent, which he told me he had used with great success in treating gonorrhœa.

The quinine was prescribed in the strength of two and a half grains to the ounce of distilled water, to be injected three or four times a day. This treatment in my hands has been followed by such uniformly good results, that I have rejected all others in its favor; and the number of cases in which I have used it has been sufficient to afford a fair test of its value.

Though the formula contained other ingredients—zinc sulphate, tincture of gelseminum, and enough sulphuric acid to dissolve the quinine--I now think its efficiency is chiefly due to the quinine, and this through the peculiar influence which the drug is now known to exert over suppuration, in checking the amœboid motion of the white blood-cells and constringing the coats of the capillaries. I lately commenced a series of experiments, putting this theory to the test.

At this time I had under treatment a patient with empyema of nearly four months' standing, whose chest I had tapped a few days before, pumping away about three pints of very fetid pus. The treatment up to this time had been a daily washing out of the cavity with a weak solution of carbolic acid; and as yet, the pus continued to be discharged in considerable quantity, and to be very offensive.

This case I thought would afford an excellent opportunity for testing the property of quinine referred to above, as well as another, that of staying the decomposition of animal matter by very effectually destroying the vitality of certain organisms upon which this decomposition seems to depend. Accordingly, every day, after cleansing the cavity with carbolic acid lotion, about six grains of quinine in two or three ounces of water were thrown in, and allowed to remain. Since the commencement of this treatment the discharge of pus has rapidly diminished, and no unpleasant odor has been noticeable. The patient in the meantime has made good progress towards recovery, and her improvement in every particular seemed to begin sharply at the time she was put on the quinine treatment.

This patient is a young mulato woman, and when admitted to the hospital was so extremely emaciated, so low in every way, that in operating I feared I should uselessly inflict pain, and must confess that I was finally moved to operate rather by the desire of Dr. Chandler, the resident physician, to see his diagnosis of empyema positively verified, than by any hope I had of giving permanent relief.

With the view of getting better drainage, I made another opening after a few days, near the most dependent part of the pleural cavity, and inserted a tube. This tube, however, slipped out, and the opening closed, leaving the first, in which a tube about a fifth of an inch in calibre had been inserted, to do the whole work.

I mention this because the patient has good prospects of recovery under a condition which has been supposed unfavorable—that of having only one small opening into the cavity; either two openings, or one made very large, by removal of a section of rib, having been deemed almost necessary to a good result.

My second experiment was made upon a large ulcer of two years' standing, situated on the leg of a woman about forty years of age, who had for some time been suffering with mitral lesion, which had already begun to cause œdema of the feet and ankles. The woman, moreover, was ill-nourished, and had a very unhealthy look. I give these particulars to show the unpromising character of the case.

The quinine was applied incorporated in benzoated zinc oxide ointment; ten grains to the ounce. The ulcer at the time this treatment was commenced secreted pus very copiously. After two or three days the suppuration had greatly diminished, and the whole surface of the sore was covered with healthy granulations. The healing process, considering the very unfavorable conditions, advanced rapidly up to the last time I saw the patient, a few days ago.

My third experiment was made in a case of mammary abscess which occurred in a primipara a few days after her delivery. The breast was opened in due time, and some six ounces of pus evacuated. Small purulent deposits, however, unconnected with each other, continued to form in spite of both general and local treatment. This condition of things seemed disposed to become chronic, and I determined to try the topical quinine treatment. The quinine, in the strength of ten grains to the ounce of water, was daily injected into she sinuses left after evacuating the small abscesses mentioned.

After being under this treatment three or four days, the deposits of pus ceased to form, and the patient rapidly got well.

As regards the gonorrhœa cases treated with quinine, I am strongly inclined to attribute the good results to the use of this agent. The average length of time required to completely arrest the discharge has been shorter under this treatment than any I have ever tried; and in no instance has a patient come back to me, after leaving off the treatment a day or two, with a return of the discharge, as so often is the case after treatment by astringents used alone. In no case has there occurred the complication of swelled testicle, a condition supposed by some, and no doubt correctly, to be due to the use of strong astringent injections; nor has any case terminated in gleet.

It may be well to add further, that I adopted this treatment in the first instance simply upon the recommendation of a very intelligent practitioner, and that I did not have a theory of its action until long after I had recognized its efficiency.

I can easily believe that one opposed to this theory of the action of quinine might not concur with me in the estimate I place upon the data derived from the other experiments, but I do not think any one free from bias could see what passed under my observation without being very favorably impressed with the merits of the treatment tested, and disposed to make further trial of it, with almost expectation of success—which, by-the-bye, is just the mental attitude I hold, after my limited experimentation.

I call attention particularly to the immediate effect following the quinine treatment upon the ulcer, of diminishing the secretion of pus, and inaugurating at once an active healing process, in which was afforded a clear illustration of the power of this agent to check the waste of force and material involved in profuse suppuration, and to direct them to the work of building healthy tissue.

Of my experiment upon the mammary abscess I wish to say that, standing alone, I regard the data gathered from it as of very little value, and only give it place as supplementary to the others.—*The Practitoner.*

